# Ancient foe spectra: Case series of Mycobacterium tuberculosis presentations

**DOI:** 10.1002/ccr3.3616

**Published:** 2020-12-05

**Authors:** Wael Goravey, Gawahir A. Ali, Mahir Petkar, Adham Ammar, Mouhammad Sharaf Eldean, Muna A. Al Maslamani, Hamad Abdel Hadi

**Affiliations:** ^1^ Department of Infectious Diseases Communicable Diseases Centre Doha Qatar; ^2^ Department of Laboratory Medicine and Pathology Hamad Medical Corporation Doha Qatar

**Keywords:** appendicitis, chest wall abscess, ganglion, polymerase chain reaction, tuberculosis

## Abstract

Extrapulmonary tuberculosis frequently eludes assessment through atypical presentations and constitute diagnostic challenges. High degree of suspicion with aids of GeneXpert MTB/RIF can clinch the diagnosis and avoid unnecessary consequences.

## Graphical abstract

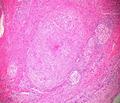

## 1 INTRODUCTION

The atypical presentations of extrapulmonary TB necessitate a high degree of suspicion. It can imitate many infectious and noninfectious conditions confounding amateurs and experts leading to morbidity and mortality. We report three different presentations of rare extra‐pulmonary tuberculosis emulating common conditions and discuss assessment, clinical course, and management.

Mycobacterial tuberculosis (MTB) remains one on the principal infections worldwide with incidence of 10.0 million cases in 2018. The prevalence of extrapulmonary varies between 8% and 24%, depending on baseline demographic characteristics, geographical locations as well as underlying host immunological status.^1^ Extrapulmonary TB can affect any viable tissues frequently raising diagnostic challenges. The most frequent affected sites outside the lungs are the lymphatic system, intestine, genitourinary tract, and central nervous as well as musculoskeletal systems.^2^

We report three rare and interesting cases of extrapulmonary tuberculosis presented to our clinical services, the diagnosis of TB was established through appropriate TB molecular methods, cultures in addition to histopathological examinations. All three cases required surgical interventions to establish diagnoses. Surgical drainage of an anterior chest wall TB abscess resembling pyogenic infection, appendectomy for an acutely inflamed appendix confirmed as localized intestinal TB, while surgical excision of ganglion‐like lesion confirmed as TB. All cases were treated with appropriate standard quadruple TB medications with uneventful outcomes.

## 2 CASES DESCRIPTION

### 2.1 Case (1)

**Figure 1:**
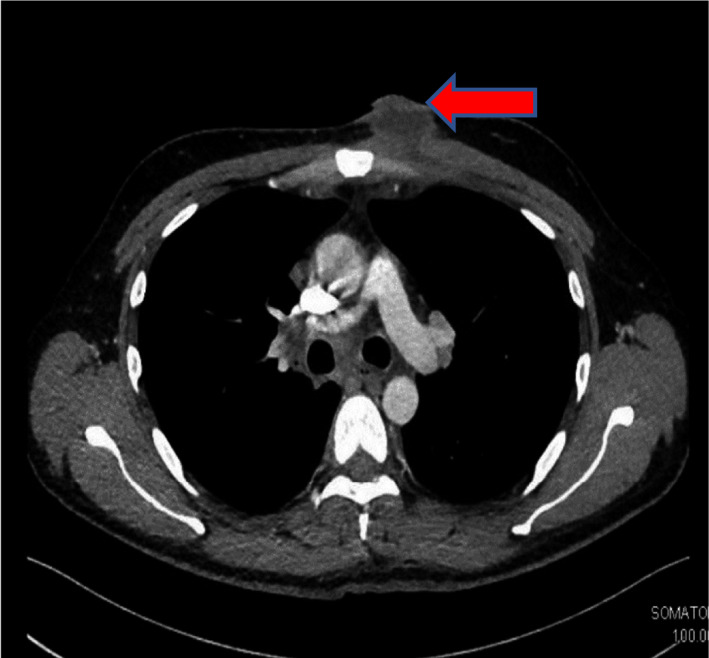
CT chest demonstrating left anterior chest wall abscess with thickened walls (arrow) and clear lung fields

A 32‐year‐old Indian male manual worker with no known past medical history, presented to our emergency department with a two weeks history of nontraumatic anterior chest wall painful swelling associated with recurrent fever. There were no other apparent significant physical signs apart from documented fever of 38°C and 4 × 4 cm fluctuant abscess at the medial aspect of the right nipple discharging purulent fluid. Blood tests were within normal limits apart from an elevated CRP of 120 mg/L (0‐5). Chest X‐ray was normal. Following assessment, the abscess was drained, and subsequent bacterial cultures were sterile. A week later, the patient represented with recurring fever as well as reaccumulation of the collection. The atypical presentation promoted reevaluation with CT chest which showed a sizable subcutaneous abscess just anterior to medial aspect of pectoralis muscle with clear lung fields (Figure 1). Surgical drainage was repeated but was processed this time for TB to widen working diagnoses. MTB (GeneXpert MTB/RIF) was positive with negative Rifampicin resistant gene and subsequently fully sensitive mycobacterial tuberculosis was isolated while HIV test was negative. The patient was treated with 6 months of standard TB therapy with no further recurrence.

### 2.2 Case (2)

**Figure 2:**
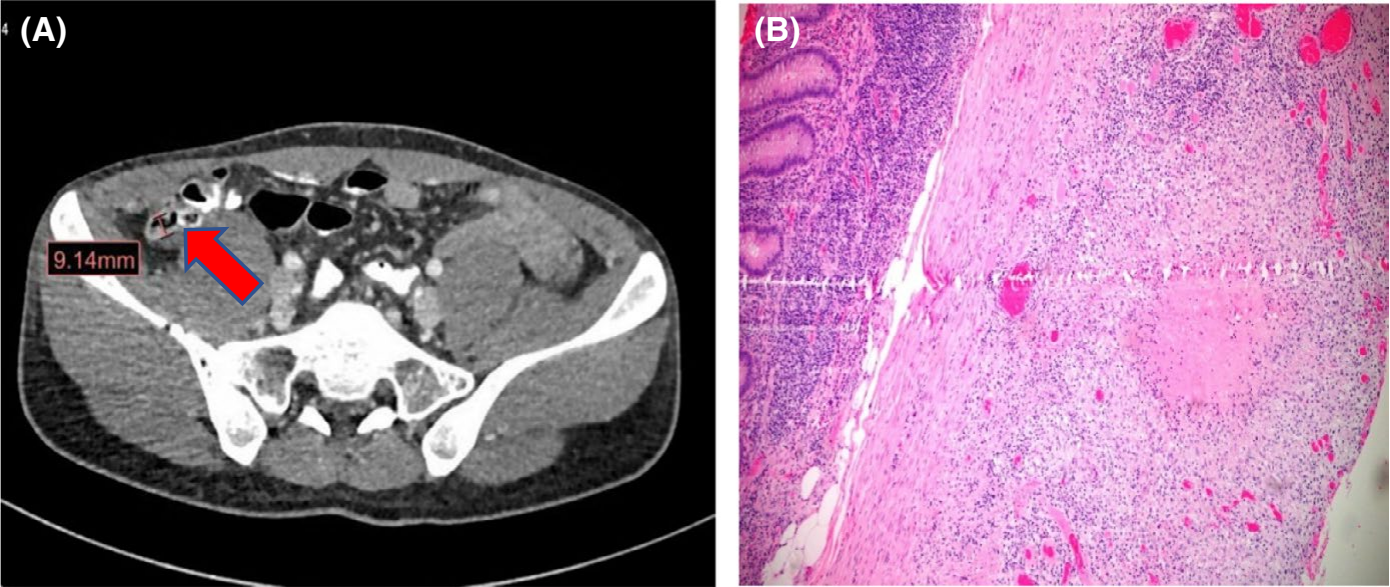
(A) CT pelvis highlighting thickened inflamed appendix (arrow) showing intraluminal necrotizing granulomatous inflammation in histopathology, B

A 29‐year‐old healthy Bangladeshi gentleman admitted with an acute history of nonradiating right lower quadrant pain for 2 days duration. There was no history of fever or other associated gastrointestinal symptoms. Rebound tenderness was detected at the affected site while initial laboratories workup demonstrated CRP of 73 mg/L (0‐5) and total leukocyte count of 13 000 (4.5‐11.0 × 109/L) with 89% neutrophils. To investigate the underlying pathology, CT abdomen with contrast showed thickened appendix, mural enhancement with intraluminal air while oral contrast demonstrated peri‐appendicular fat stranding suggestive of appendicitis (Figure 2A).

The patient was admitted for suspected appendicitis when subsequent laparoscopic appendectomy demonstrated inflamed appendix but with distinctive multiples white yellowish lesions at the base of the inflamed appendix not noticeable elsewhere raising the suspicion of intestinal TB. Samples from biopsied lesions were positive by TB PCR with no associated resistance while subsequent culture from obtained tissues grew fully sensitive MTB. Histopathological examination showed necrotizing granulomatous inflammation consistent with TB (Figure 2B). The patient responded well to standard 6 months course of anti‐TB medications with no recurrence upon follow‐up.

### 2.3 Case (3)

**Figure 3:**
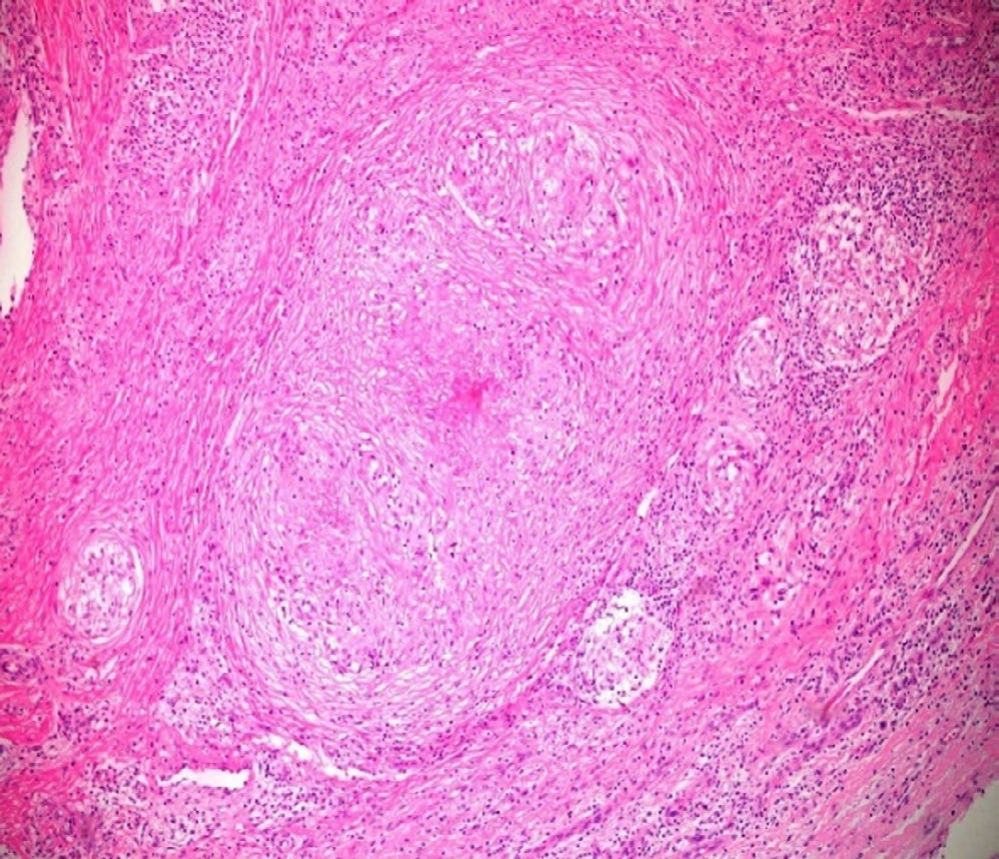
Histological examination of excised soft tissues demonstrating intense inflammation with a granuloma

A 28‐year‐old right‐handed Bangladeshi male, referred to the hospital because of a two‐month history of nonpainful swelling at the dorsum of the right hand. There was no significant past medical history of note nor a recent history of trauma. Examination showed a nontender multinodular transluminal swelling on the dorsal aspect of right wrist, measuring around 3 × 4 cm with laxity and gliding of the overlying skin. The hand function was preserved with good hand grip of grade 5 and good range of joint movement. Initial laboratory tests were within normal limits while X‐ray of the hand showed no bony involvement. The initial impression was of a lipoma or a complicated ganglion swelling. Complete excision of the lesion was performed when intraoperative findings demonstrated an ill‐defined fibro‐fatty lesion adherent to the wrist carpal tendons sheaths extensors with thickening of the synovium probably originating from the wrist joint. Histopathology of the excised tissues showed necrotizing granulomatous inflammation (Figure 3) while microbiological cultures were negative, however, TB PCR was positive with no accompanied resistance. Subsequent radiological imaging demonstrated no pulmonary involvement while HIV test was negative. Based on obtained results, the patient was commenced on standard 6 months TB therapy confirmed on subsequent cultures as fully sensitive MTB. The patient had no recurrence one year into follow‐up.

## 3 DISCUSSION

Extrapulmonary TB is capable of affecting all sites in the human body when frequently it might mimic inflammatory, infectious, or neoplastic conditions.^3^ Despite observed challenges during the assessment of extrapulmonary tuberculosis, the diagnoses can be established readily provided the potential possibility is considered while evaluating working diagnoses. During systematic patients’ assessment, appropriate history might reveal disease chronicity as opposed to acute, typical of disease presentation. Conversely, progressive persistent symptoms that do not respond to other alternative management or failure to isolate pathogens repeatedly by obtaining sterile cultures should raise the suspicions of indolent pathogens such as TB demonstrated in the first presented case.^4^

Anterior chest wall TB is rare, representing around 1%‐2% of all TB cases.^5^ The main modes of establishing the underlying pathology are hematogenous spread, direct inoculation, nt was obtai or extension from adjacent infected tissues.^6^ Our case highlights chest wall TB might frequently resemble pyogenic infections unless hints of MTB infections are considered such as previous history of pulmonary TB, TB exposure, recurrent or indolent presentation with negative initial cultures.

For suspected cases, radiological imaging preferably CT is a crucial step during initial assessment particularly to rule out potential muscles or bone extensions as well as in excluding simultaneous pulmonary involvements.^5^, ^6^ The diagnosis is confirmed early through molecular methods with TB PCR, otherwise, awaiting the results of TB cultures or obtaining histopathological samples are sometimes needed.^7^ The mainstay of the management of TB‐associated abscesses is combined surgical excision particularly if there are large local involvement, followed by standard medical TB therapy.^8^

Notably, gastrointestinal tuberculosis accounts for 1%‐3% of all extrapulmonary TB forms while tuberculous appendicitis is extremely rare cited as only 0.1%‐0.6% of the cases.^9^ The exact pathogenesis is not fully understood but hematogenous spread or extension from neighboring organs is the likely implicated source. Characteristically, intestinal TB is frequently associated with abnormal findings during laparotomy or laparoscopic exploration of secondary ascites, adhesions typical yellowish/whitish lesions suggestive of TB as our case.^10^ Because of similarities of clinical presentation to typical appendicitis, in most of cases tuberculous pathology can only be suspected intraoperatively or upon subsequent histological examination.^11^ This case also highlights the emerging role of molecular test like TB PCR in early diagnosis and confirmation of intestinal TB.^7^ Reportedly, GeneXpert performance in intestinal TB yield sensitivity of 95.7% when compared with cultures (35.0%), and both achieved 100% specificity.^12^ The third case highlights one of the ramifications of musculoskeletal TB. Although tuberculosis of the hand is seen in 10% of musculoskeletal TB, ganglion‐like TB of the wrist and hand is rare with few reported cases in the literature.^13^, ^14^, ^15^, ^16^ The clinical picture as our case is indistinguishable from other similar pathology and only confirmed upon histopathological examination.^13^ Assessment of hand TB entails appropriate imaging to examine extend of the disease particularly MRI, which is the best for joint and soft tissues assessment, and simple aspiration for microbiological identifications.^17^ The reported TB PCR specificity from ganglion samples was 64% in compare with 100% in others osteoarticular samples, while 64.3% sensitivity was reported from pus sample.^18^ Besides surgical excision, intraoperative tracking of disease extension facilitates exploring extension into deep tissues.^17^ Treatment follows recommended standard six months therapy.

These atypical presentations of extrapulmonary TB necessitate high degree of suspicion starting with reasonable doubts in affected populations particularly among immigrants from high burden endemic countries, coupled with systematic approaches of suspecting infections as a potential assessment for unusual presentation which entails processing specimens for needed investigations.

## 4 CONCLUSION

Tuberculosis remains a fascinating disease both in the spectra of pathology and variety of presentations. It can imitate many infectious and noninfectious conditions confounding amateurs and experts. During patients’ evaluation, high index of suspicion is needed since the disease cannot be established or confirmed unless suitable routes are sought, and the appropriate tests are requested. Once considered, establishing or refuting potential possibilities can be then easily evaluated to avoid the unnecessarily consequences particularly delays in timely management.

## CONFLICT OF INTEREST

The authors declare that they have no competing interests.

## AUTHOR CONTRIBUTIONS

WG: involved in corresponding author, data acquisition, and manuscript preparation. GA: contributed to data acquisition and manuscript writing. MP, AA, and MS: contributed to data acquisition and histopathology reports. HA and MA: supervised all the aspects and contributed to final manuscript editing.

## Data Availability

The authors confirm that the datasets supporting the findings of this case are available from the corresponding author upon request.
